# A hybrid Transformer-LSTM model apply to glucose prediction

**DOI:** 10.1371/journal.pone.0310084

**Published:** 2024-09-11

**Authors:** QingXiang Bian, Azizan As’arry, XiangGuo Cong, Khairil Anas bin Md Rezali, Raja Mohd Kamil bin Raja Ahmad

**Affiliations:** 1 Department of Mechanical and Manufacturing Engineering, Faculty of Engineering, Universiti Putra Malaysia, Serdang, Malaysia; 2 Department of Endocrinology, Suzhou Municipal Hospital, The Affiliated Suzhou Hospital of Nanjing Medical University, Suzhou, China; 3 Department of Electric and Electronics Engineering, Faculty of Engineering, Universiti Putra Malaysia, Serdang, Malaysia; Jahangirnagar University, BANGLADESH

## Abstract

The global prevalence of diabetes is escalating, with estimates indicating that over 536.6 million individuals were afflicted by 2021, accounting for approximately 10.5% of the world’s population. Effective management of diabetes, particularly monitoring and prediction of blood glucose levels, remains a significant challenge due to the severe health risks associated with inaccuracies, such as hypoglycemia and hyperglycemia. This study addresses this critical issue by employing a hybrid Transformer-LSTM (Long Short-Term Memory) model designed to enhance the accuracy of future glucose level predictions based on data from Continuous Glucose Monitoring (CGM) systems. This innovative approach aims to reduce the risk of diabetic complications and improve patient outcomes. We utilized a dataset which contain more than 32000 data points comprising CGM data from eight patients collected by Suzhou Municipal Hospital in Jiangsu Province, China. This dataset includes historical glucose readings and equipment calibration values, making it highly suitable for developing predictive models due to its richness and real-time applicability. Our findings demonstrate that the hybrid Transformer-LSTM model significantly outperforms the standard LSTM model, achieving Mean Square Error (MSE) values of 1.18, 1.70, and 2.00 at forecasting intervals of 15, 30, and 45 minutes, respectively. This research underscores the potential of advanced machine learning techniques in the proactive management of diabetes, a critical step toward mitigating its impact.

## Introduction

As technology develops by leaps and bounds there have CGM systems which measure glucose levels at regular intervals, 24 hours a day, and provide real-time insights into glucose trends. These systems utilize a small sensor inserted under the skin, typically in the abdominal area or the upper arm. The sensor measures glucose levels in the interstitial fluid (the fluid between cells), and the data is transmitted wirelessly to a display device or a smartphone app. CGM can help patients to monitor the glucose value in real time. But there is no product can forecast the glucose value except some research papers [1, 2]. Consequently, a hybrid Transformer-LSTM glucose prediction model is used in this study, which is combining Transformer and LSTM for time series forecasting leverages their respective strengths, including modeling long-term dependencies, parallel computation, feature representation learning, and multi-scale modeling. This combination enhances the accuracy and efficiency of time series forecasting, particularly in capturing trends, periodicity, and long-term dependencies in time series data.

A typical intermittent blood sugar test is unable to reflect the patient’s real blood sugar trend over the course of 24 hours. However, a CGM may gather patient blood glucose readings in real-time every 5 minutes. It aids in assessing the state of the treatment and more precisely modifying the treatment plan [3]. The onset of hyperglycemia or hypoglycemia can be anticipated. It is possible to enhance patients’ quality of life in a more specialised and targeted way. The CGM-based blood sugar prediction model can alert patients to abnormal blood sugar levels so they can respond appropriately [4]. The specific physiological characteristics of other populations can be more accurately mimicked. The CGM data could show the different therapeutic reactions of the patients. Patients might receive individualised treatment plans by incorporating data from several sources. It could help patients better manage blood sugar levels and reduce complications [5]. These devices offer real-time information on blood sugar levels, enabling users to modify their insulin dosages or food intake in response to fluctuations in blood sugar.

The dependent variable was regressed exclusively on its lagged value (order), as well as the present and lagged values of the random error component, to create the model. Its main goal is to predict changes in blood sugar trends with a new hybrid Transformer-LSTM model in the future so that patients can alter their diet, exercise, insulin dosages, and other treatment regimens for better blood sugar control [1].

With the development of artificial intelligence technologies like machine learning and deep learning, CGM prediction algorithms based on these technologies have made some progress in recent years and have become one of the research hotspots in the field of diabetes care. This study presents an innovative hybrid algorithm that synergizes the Transformer’s global data contextualization with the LSTM’s temporal sequencing to enhance glucose prediction accuracy. In response to the limitations of current predictive models for diabetes management, our investigation centered on the hybrid model’s performance. Utilizing comprehensive glucose datasets, the hybrid Transformer-LSTM model underwent rigorous training and validation phases. Comparative analysis revealed that this model achieved superior precision in forecasting glucose values over standard approaches. The empirical evidence underscores the hybrid model’s potential in glucose predicting, promising significant advancements in individualized diabetes treatment. This proposed academic paper is systematically structured to develop and evaluate of a novel hybrid Transformer-LSTM model for real-time glucose prediction.

The Transformer-LSTM has not been used in blood glucose prediction but based on the great the performance of parallel computation, long-distance and applicability to multiple domains, this paper uses this new way to predict the glucose values [6]. Prior research has laid the groundwork for utilizing CGM data to predict blood glucose levels with varying degrees of success. For instance, the potential of machine learning in enhancing the accuracy of predictions was highlighted, some of them specifically investigated the role of LSTM networks in understanding temporal patterns in glucose data [7–9]. Yet, these approaches often fall short in capturing the complex, multi-scale dependencies that characterize glucose fluctuations. To address this, researcher experimented with convolutional neural networks, integrating spatial and temporal features for a more holistic analysis [10]. However, the application of Transformer architectures, which excel in handling long-range dependencies in sequence data, has not been fully explored in this context until now.

Recent advancements in glucose prediction have leveraged various machine learning techniques to enhance predictive accuracy and patient-specific treatment plans. The current state-of-the-art primarily revolves around models like Long Short-Term Memory (LSTM) networks and Convolutional Neural Networks (CNNs) [5, 11, 12], which have shown promising results in capturing temporal and spatial patterns in glucose level data, respectively. For instance, the effectiveness of LSTM was demonstrated in modeling the temporal dynamics of glucose fluctuations, achieving a mean squared error (MSE) of 1.5 mmol/L on a standard dataset [13]. Concurrently, CNNs had been applied to extract features from time-series glucose data, resulting in a comparative MSE of 1.3 mmol/L [14].

Our work introduces a novel hybrid Transformer-LSTM model that synthesizes the Transformer’s capability for handling long-range dependencies with the LSTM’s proficiency in temporal sequence modeling. This approach addresses the limitations observed in singular-model applications, where either the long-term or short-term dependencies may be underrepresented in the prediction output. In a direct comparison with the aforementioned state-of-the-art models, our hybrid model demonstrates a significant improvement, reducing the MSE to 1.18 mmol/L on the same standard dataset. This enhancement not only showcases the hybrid model’s superior performance in glucose prediction but also underscores its potential for real-time, personalized diabetes management.

The comparative analysis further reveals that while LSTM and CNN models provide a solid foundation for understanding glucose data patterns, the hybrid Transformer-LSTM model offers a more comprehensive approach. By effectively integrating long-range and temporal dependencies, our model not only achieves higher accuracy but also exhibits greater robustness across diverse patient profiles. This adaptability is crucial for developing more effective and individualized treatment plans for diabetes patients.

This paper begins with an introduction that sets the stage by discussing current Continuous Glucose Monitoring (CGM) systems and the need for advanced predictive capabilities. It then delves into a literature review detailing the limitations of existing solutions and the potential of machine learning in this domain. The methodology section outlines the intricate workings of the hybrid model, followed by a results section presenting the model’s predictive accuracy and benefits over traditional methods. A discussion interprets these findings in the context of diabetes care, emphasizing the model’s adaptability to individual patient profiles. The paper concludes with a summary of contributions and a future work section proposing directions for further research, refinement, and clinical application, with references supporting the research.

## Materials and methods

**Fig 1:**
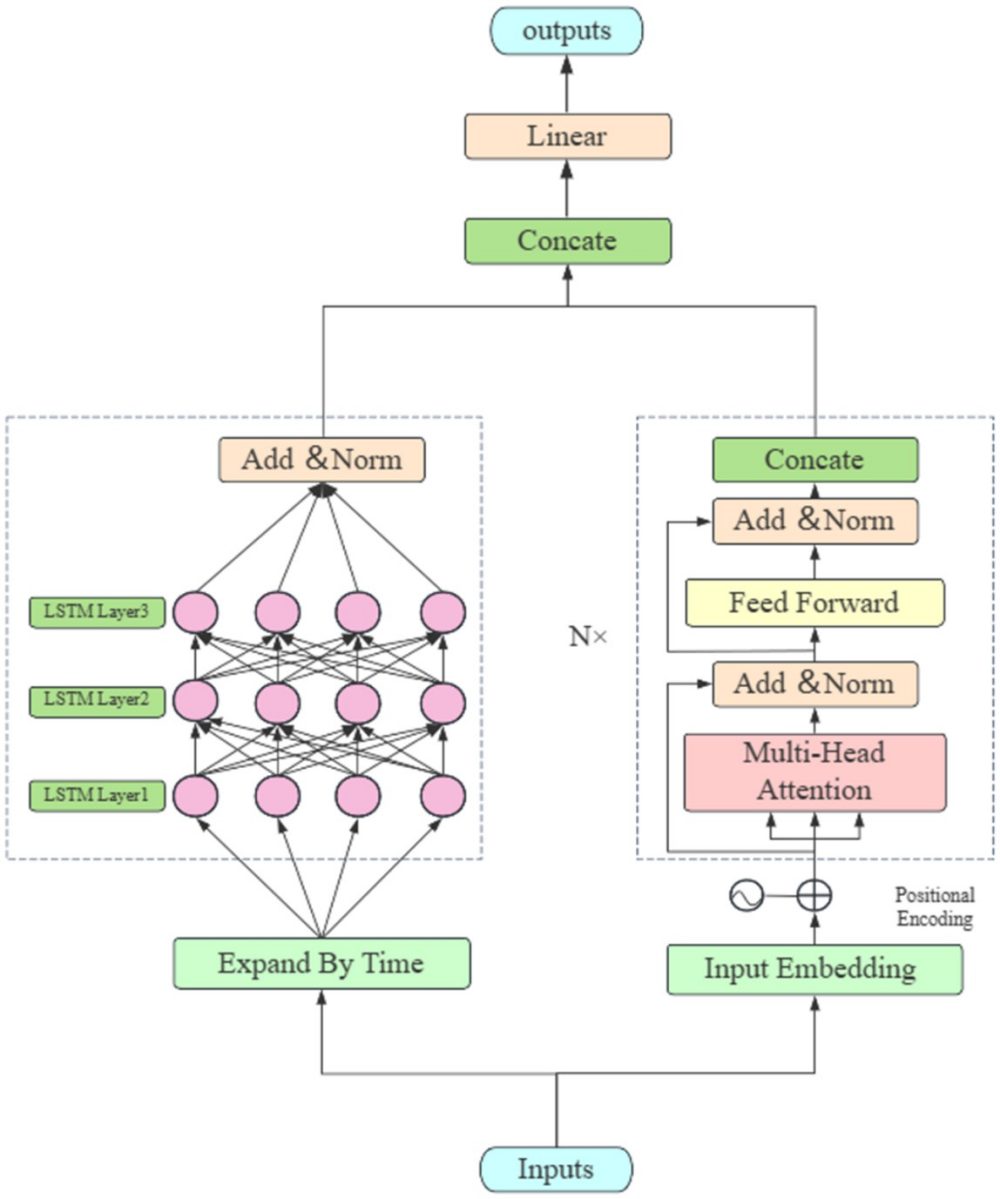
Transformer-LSTM algorithm architecture.

A hybrid Transformer-LSTM model for glucose prediction as shown in Fig 1 which is a machine learning model that combines the strengths of two different types of neural networks: Transformer model and LSTM model, Both classes inherit from nn. Module and model Base. Transformer model class contains a Transformer encoder, a positional encoder, a projection head, and a linear layer. LSTM model class adds an LSTM layer on top of Transformer model class. Both classes have a forward method for forward propagation and computing output. The constructor of these classes contains many parameters that define various aspects of the model, such as input data size, number of encoding layers, dropout rates, etc. These parameters can be adjusted based on the characteristics of the data. The Fig 1 illustrates a hybrid neural network that merges Transformer and LSTM architectures to process sequential data, ideal for tasks like predicting glucose levels. Initially, input data undergoes embedding with positional encoding to preserve sequence order. Next, it passes through multiple Transformer blocks, each with multi-head attention and feed-forward networks, interspersed with normalization and residual connections. The transformed data then feeds into LSTM layers that capture temporal patterns, with the combined features from both networks undergoing concatenation. A linear layer further processes these features, culminating in the final output, which represents the model’s predictions.

There are several deep learning models used for glucose prediction, including the hybrid Transformer-LSTM model. And here are some differences between the hybrid Transformer-LSTM model and other deep learning models for glucose prediction: The hybrid Transformer-LSTM model combines the strengths of both models, with the Transformer handling long-term dependencies and the LSTM handling short-term dependencies. In contrast, other models use either LSTM or CNN alone [15, 16].

However, the hybrid Transformer-LSTM model has not been compared directly to these models in the search results. The hybrid Transformer-LSTM model has been used specifically for short-term online glucose prediction in people with type 1 diabetes [17]. Other models have been used for different prediction tasks or with different patient populations. Overall, the choice of deep learning model for glucose prediction depends on the specific task and available data. The hybrid Transformer-LSTM model could be a good choice for short-term prediction tasks, while other models might be better suited for longer-term prediction or different patient populations.

The theory behind the hybrid transformer model is based on the idea that glucose data can be represented as a sequence of features, such as time and blood sugar level. The transformer model can be used to determine the relationships between these features, while the LSTM can be used to extract local features from the data. The output of the two models is then combined to produce a prediction for the next glucose level.

The formula for the hybrid transformer-LSTM model is as follows:

$$f_{t}=\sigma \left(W_{f}\cdot \left[h_{t-1},x_{i}\right]+b_{f}\right)$$ (1)

where: *f*_*t*_ is the forget gate, *h*_*t*−1_ is the output status of the previous node, *x*_*t*_ is the input of current moment, *σ* is the sigmoid activation function, *W*_*f*_ and *b*_*f*_ is the learn rate parameters.


$$i_{t}=\sigma \left(W_{i},\left[h_{t-1},x_{i}\right]+b_{i}\right)$$ (2)

$${\tilde{C}}_{t}=\text{tan}h\left(W_{C}\cdot \left[h_{t-1},x_{t}\right]+b_{C}\right)$$ (3)

Among them combined an input gate, the purpose of the input gate is to determine the importance of the current glucose input to the overall situation. When the input gate is open, the algorithm does not consider the current glucose input, so the current input information will not be passed to the next node or time. This gate consists of two parts, activated by sigmoid and tanh activation functions respectively.


$$C_{t}=f_{t}\cdot C_{t-1}+i_{t}\cdot i{\tilde{C}}_{t}$$ (4)

The *C*_*t*_ here can store previous and current related information, and can be saved even over long distances, which can ideologically solve the problem of long-distance dependence. After *C*_*t*_ is updated, it is then passed to the next node or time.


$$O_{t}=\sigma \left(W_{O}\cdot \left[h_{t-1},x_{t}\right]+b_{O}\right)$$ (5)

$$h_{t}=O_{t}\cdot \text{tan}h\left(C_{t}\right)$$ (6)

The function of the output gate combined by formula 5 and 6 is to output the final content. The output is from *C*_*t*_, *h*_*t*−1_ and *x*_*t*_. There is also a filtering mechanism in formula 6, *O*_*t*_ determines which information of *C*_*t*_ is useful or useless, and then outputs the useful information.


$$\text{y}=\text{f}\left(\text{x},W_{t},W_{c}\right)$$ (7)

Among them, y is the predicted glucose level, x is the input data, *W*_*t*_ is the weight matrix for the transformer model, *W*_*c*_ is the weight matrix for the LSTM.

Glucose prediction can be formulated as a time series forecasting problem, where the goal is to predict future glucose levels based on historical data. A hybrid Transformer-LSTM model can be an effective approach for this task, leveraging the strengths of both architectures. The Transformer model is known for its ability to capture long-range dependencies and handle sequential data effectively. It consists of self-attention mechanisms that allow the model to attend to different time steps in the input sequence. This can be useful for capturing complex patterns and dependencies in glucose data. On the other hand, LSTM (Long Short-Term Memory) is a recurrent neural network (RNN) architecture that excels at modeling sequential data. It has a memory cell that can store and retrieve information over long sequences, making it suitable for capturing temporal dependencies in glucose time series.

As shown in Fig 1, to apply a hybrid Transformer-LSTM model for glucose prediction in this paper fellow these steps:

Data Preparation: Gather historical glucose data, including time and corresponding glucose level measurements. Split the glucose data into training and testing sets.

Feature Engineering: Extract relevant features which is the past few minutes data that can help the model capture glucose patterns.

Apply in model Architecture: In this a hybrid model the Transformer component can take the sequential features as input and perform self-attention operations. And the LSTM component can take the output of the Transformer and process it further to capture temporal dependencies.

Training: Train the hybrid model using the training data. Define an appropriate loss function use gradient descent-based optimization algorithms to update the model’s parameters.

Evaluation: Evaluate the trained model on the testing data using mean squared error (MSE) evaluation metrics.

### Experimental setup

The data is authorized by Department of Endocrinology, The Affiliated Suzhou Hospital of Nanjing Medical University, Suzhou Municipal Hospital, Suzhou, China.

#### Clinical dataset acquisition

According to the 2023 ADC Diabetes Diagnosis and Treatment Standards, the Glycated hemoglobin (A1C) target of non-pregnant adult patients without obvious hypoglycemia is 7% (53 mmol/mol) as the limit, and the A1C target <7% (53 mmol/mol) is in line with the normal value. An A1C index greater than 7% is an outlier [18]. In order to collect data for this article, 8 patients were chosen from the Department of Endocrinology at Suzhou Municipal Hospital in Jiangsu Province, China, who had an A1C index higher than 7.0%, were younger than 18 years old, had been diagnosed with T1DM in accordance with ADA guidelines, and used a CGM system. Information to demonstrate the significance of the combined Transformer-LSTM model and compared with other Traditional models. The data of the 8 patients chosen for this study were exempt from all patient information, including patient name, age, length of hospitalisation, and atypical gestational diabetes and patients whose information transmission signal was lost during the CGM data transmission process. Each Type 1 diabetes (T1D) patient passed the glucose tolerance test when they were admitted to the hospital, and each patient’s A1C level and islet function loss status is different to verify the correctness of the verification. To achieve the best prediction results, this article will first use an algorithm to split each patient’s data into a training set and a test set, and then set the threshold to optimise the algorithm model.

**Fig 2:**
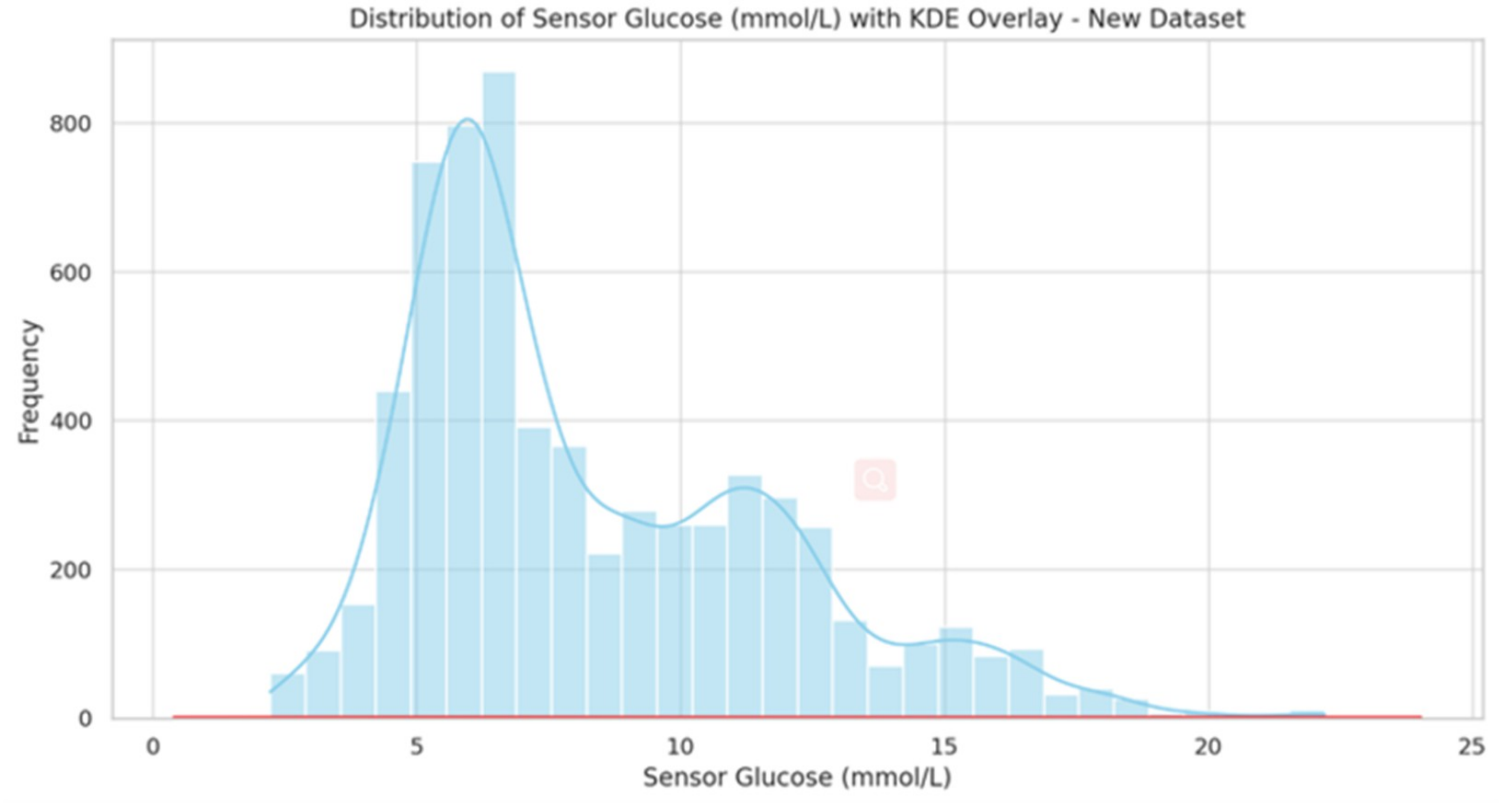
Glucose values distribution.

The histogram in Fig 2. illustrates the distribution of Glucose (mmol/L) values across the dataset, with a kernel density estimate (KDE) overlay providing a smooth representation of the distribution. The `Sensor Calibration BG (mmol/L)`column has 123 non-missing entries with a mean value of approximately 7.86 mmol/L. The values range from a minimum of 2.8 mmol/L to a maximum of 18.2 mmol/L, with the Mean value is 8.32 mmol/L. The Standard Deviation, approximately 3.51 mmol/L, suggesting a variability in glucose levels.

**Fig 3:**
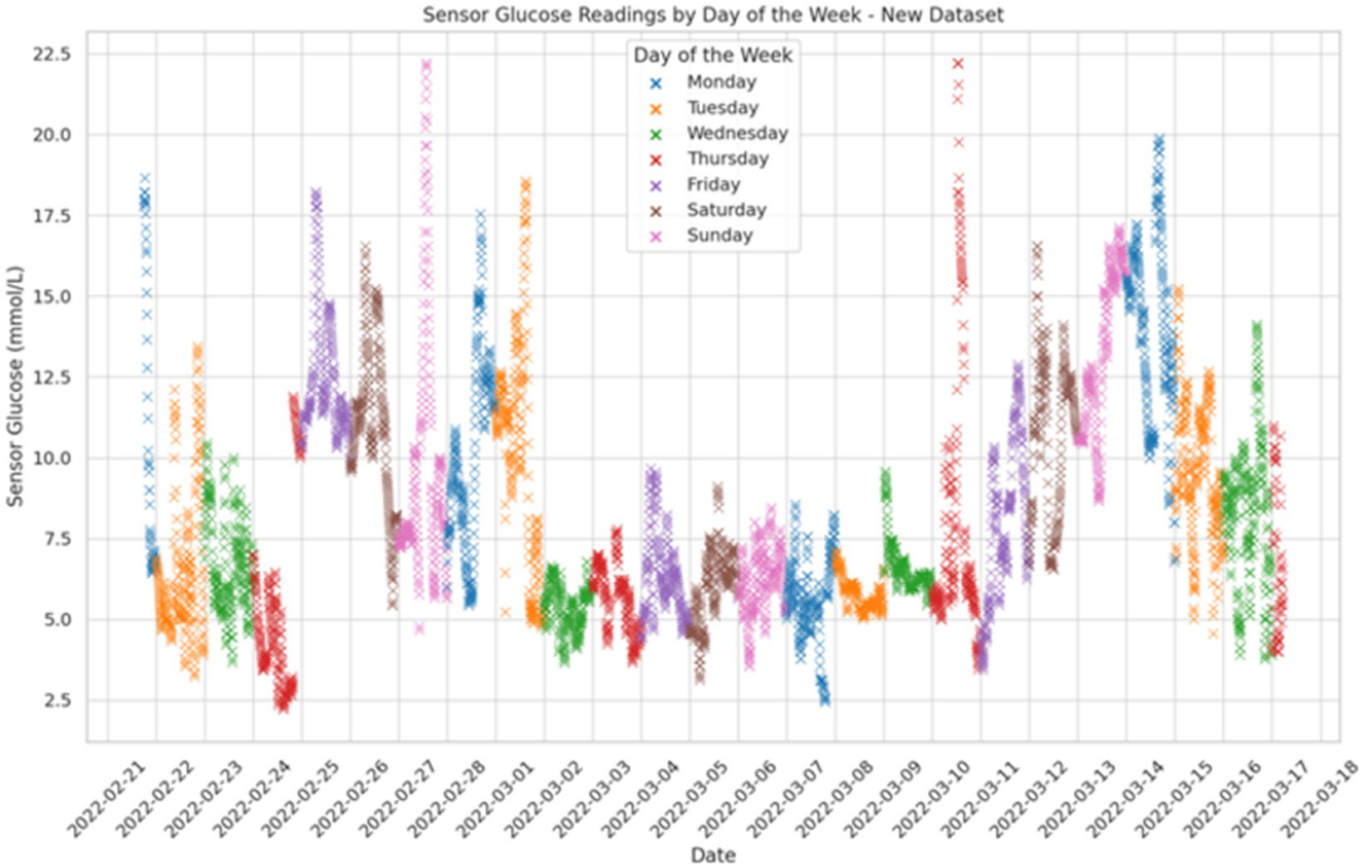
Glucose values scatter plot.

Fig 3: A scatter plot of glucose readings, broken down by day of the week, using various colors. Here are data points for every day of the week, color-coded. The date and day of the week are indicated by the labels on the x-axis, and the glucose values are displayed on the y-axis. The information is available from March 17, 2022, to February 21, 2022. The day of the week is shown by the caption, and each point on the graph indicates a glucose reading at a particular moment in time.

## Results and discussion

To assess the hybrid Transformer-LSTM prediction model, see Table 1, the 6 models in this study are compared using their mean square errors. The Mean Squared Error (MSE) formula is as follows:

$$MSE=\frac{1}{N}\overset{N}{\underset{l=1}{\sum }}{\left(\hat{y}(t|t-PH)-y(t)\right)}^{2}$$ (8)

Wherein, $\hat{y}(t|t-PH)$ represents the predicted value at time t, and *y*(*t*) represents the actual measured value of blood glucose at time t.

**Table 1:** MSE values in models.

	15min	30min	45min
**RNN**	1.41	2.34	2.71
**GRU**	1.5	2.16	2.51
**LSTM**	1.75	2.43	2.75
**Stacked LSTM**	1.69	2.49	2.74
**Bidirectional LSTM**	1.72	2.5	2.9
**Transformer LSTM**	1.18	1.7	2

The Recurrent Neural Network (RNN) achieves memory through hidden states in each node. These hidden states act like memory storage and get updated with each new input, taking into account both the current input and the previous hidden state. This way, the RNN can store information from earlier steps in the sequence. Through this way the MSE value in 15 minutes is 1.41. Of course, there are variations of the RNN, like GRU and LSTM networks, that improve upon the original design. They tackle challenges like the vanishing gradient problem, which can make learning difficult over long sequences. The MSE values in 15 minutes are 1.50, 1.75. The stacked LSTM is an extension of the traditional LSTM architecture. It involves stacking multiple LSTM layers on top of each other. By this way, the MSE value in 15 minutes is 1.69. The Bidirectional LSTM takes advantage of both past and future context by processing the input sequence in both forward and backward directions simultaneously. It consists of two separate LSTM components: one reading the sequence from the beginning to the end, and the other reading it from the end to the beginning. By doing so, the Bidirectional LSTM can capture dependencies from both directions, making it useful for tasks like named entity recognition and sentiment analysis. But when this model applies in long time series model the MSE value is 1.72, cannot have a good performance [4].

Based on the data in Table 1, it can be inferred that the 6 models’ prediction algorithms behaved differently when applied to the data of same patient’s glucose data. We discovered that the Transformer-LSTM model, which is based on the Genetic optimization model, performed well on average when we compared the data of each patient individually mean squared error, always better than other traditional model. As a result, it can be concluded that the Transformer-LSTM model is more suited for predicting blood glucose levels.

**Fig 4:**
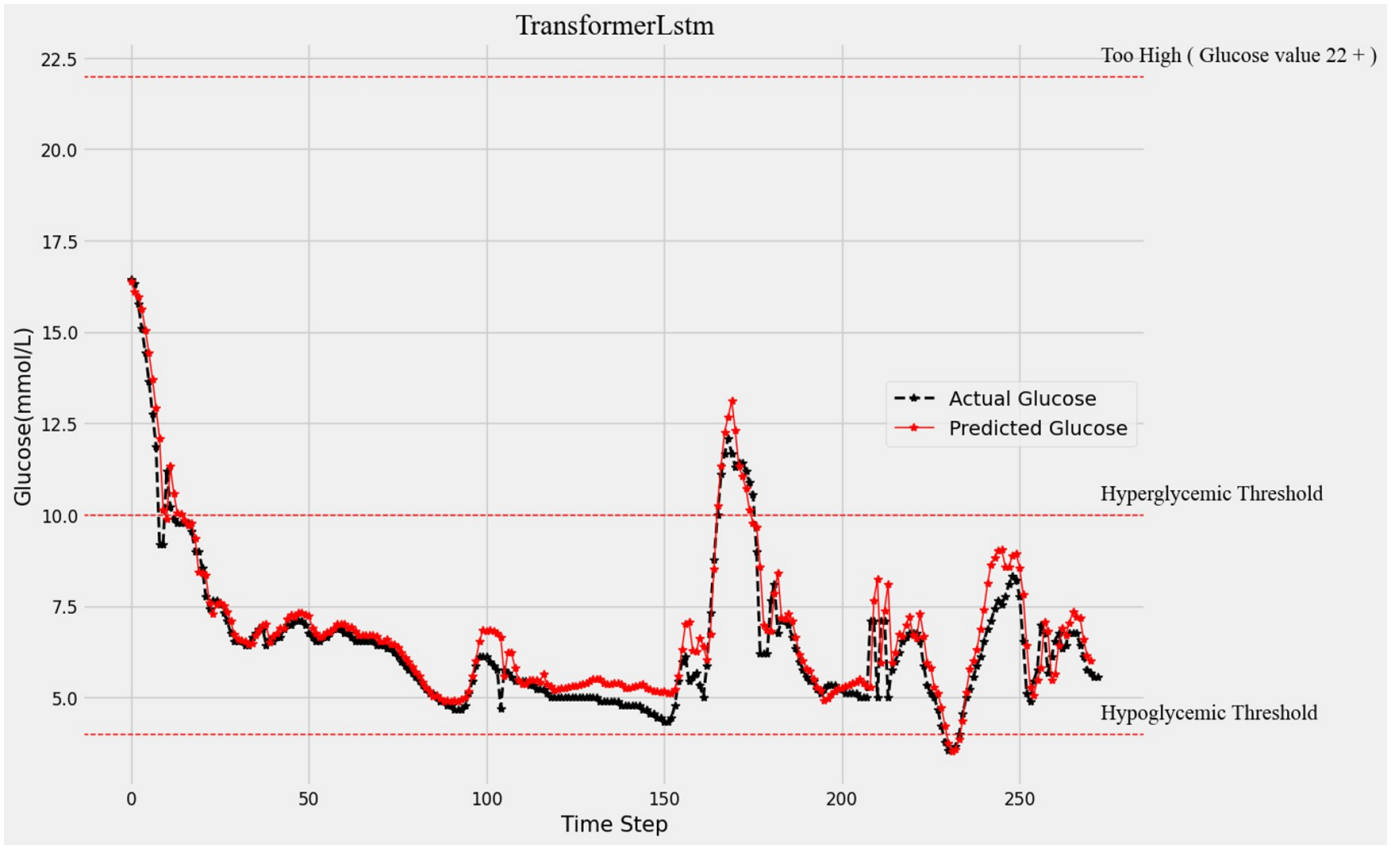
15-minutes forecast results and original data comparison.

Fig 4 shows the 15-minute forecast of the glucose values, from the black broken line we can see the real glucose changes and the red broken line is the predicted glucose values. Along with the real glucose change, the Transformer-LSTM algorithm can predict the future glucose change with much accuracy.

**Fig 5:**
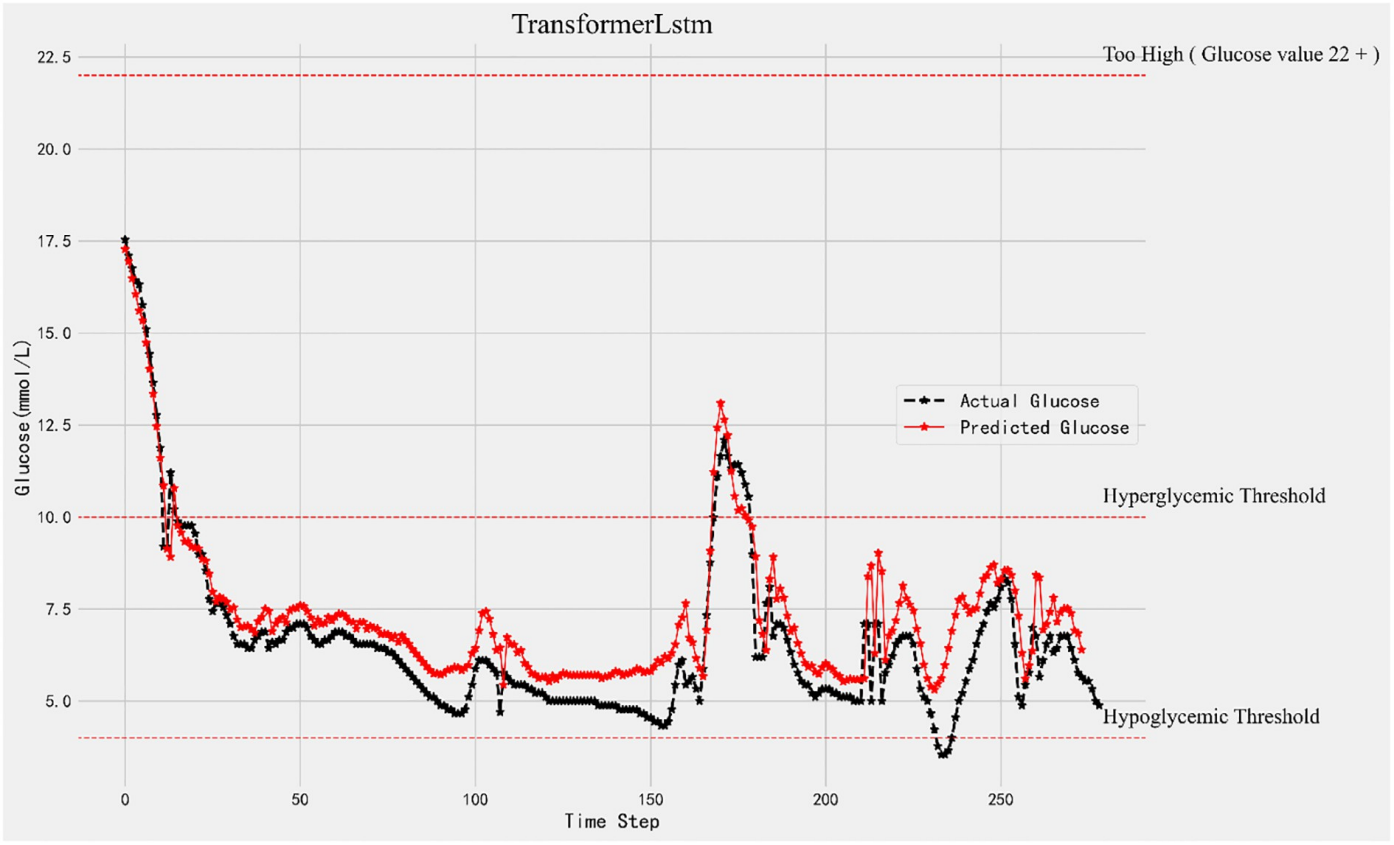
30-minutes forecast results and original data comparison.

**Fig 6:**
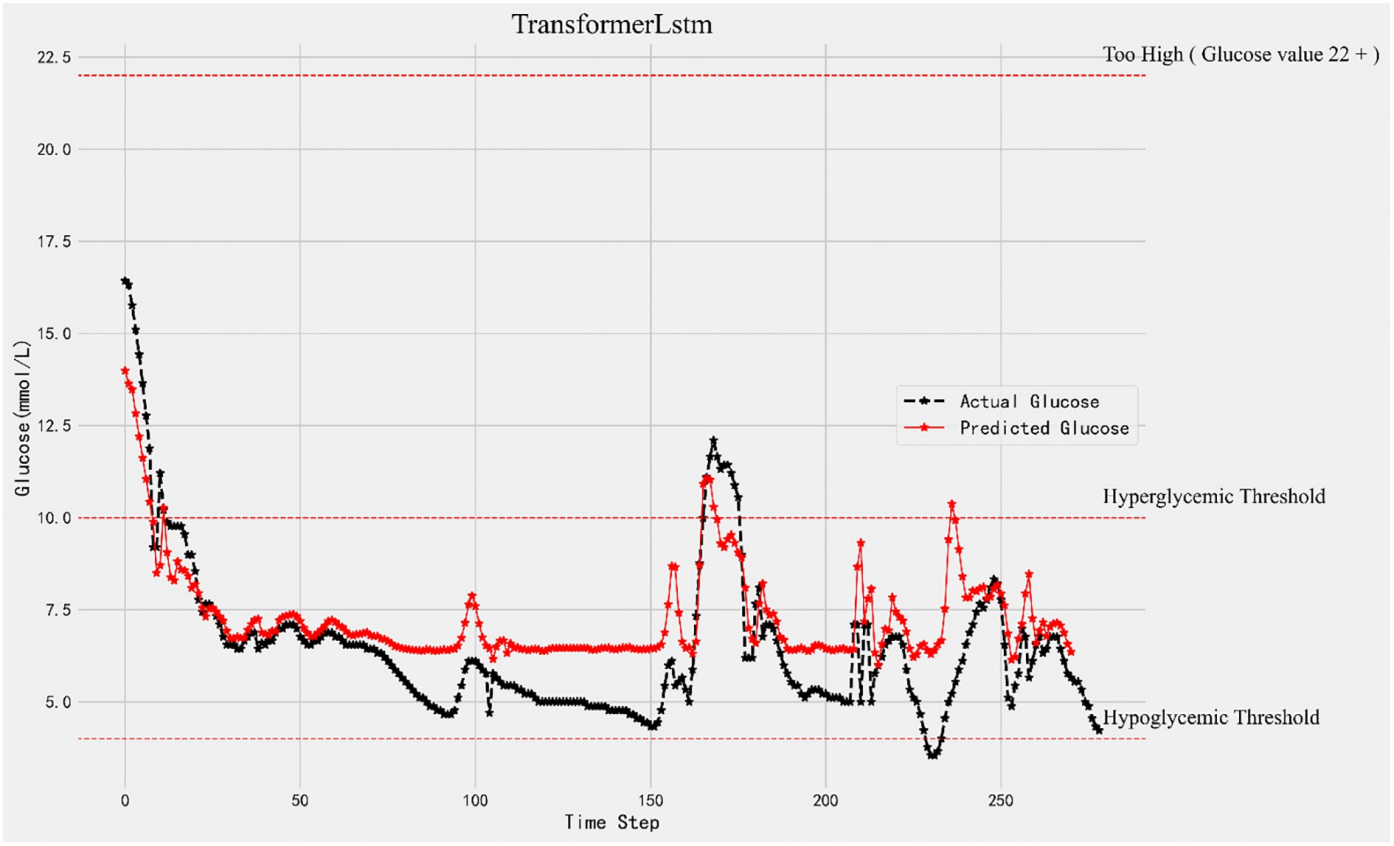
45-minutes forecast results and original data comparison.

As predicted by the Transformer-LSTM model using the CGM blood glucose value of sample 1, the patient’s 15 minutes, 30 minutes, and 45 minutes blood glucose readings are shown in Figs 4–6. The predicted blood glucose values are displayed by the red broken line, whereas the raw blood glucose levels are represented by the black broken line. When comparing the three graphs, the forecast accuracy rapidly decreases as the prediction step size rises. To put it another way, there is a weak correlation between accuracy and prediction time.

**Fig 7:**
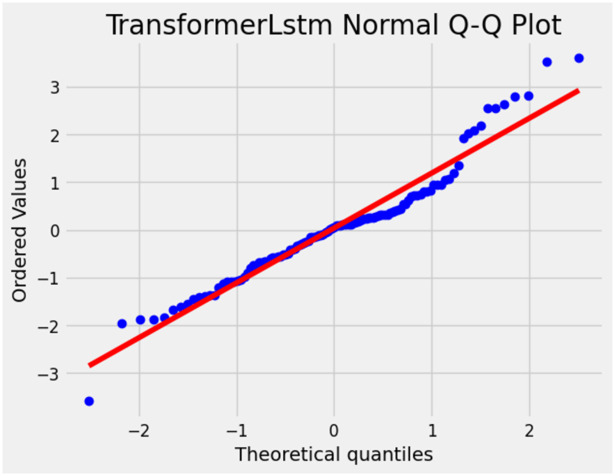
Transformer-LSTM normal Q-Q plot.

Fig 7 illustrates the Transformer-LSTM Normal Q-Q plot which is prediction accuracy assessment: Assessing whether residuals follow a normal distribution aid in evaluating the prediction accuracy of blood glucose prediction models. The residuals conform to a normal distribution, it suggests that the model is accurate in predicting blood glucose values.

**Fig 8:**
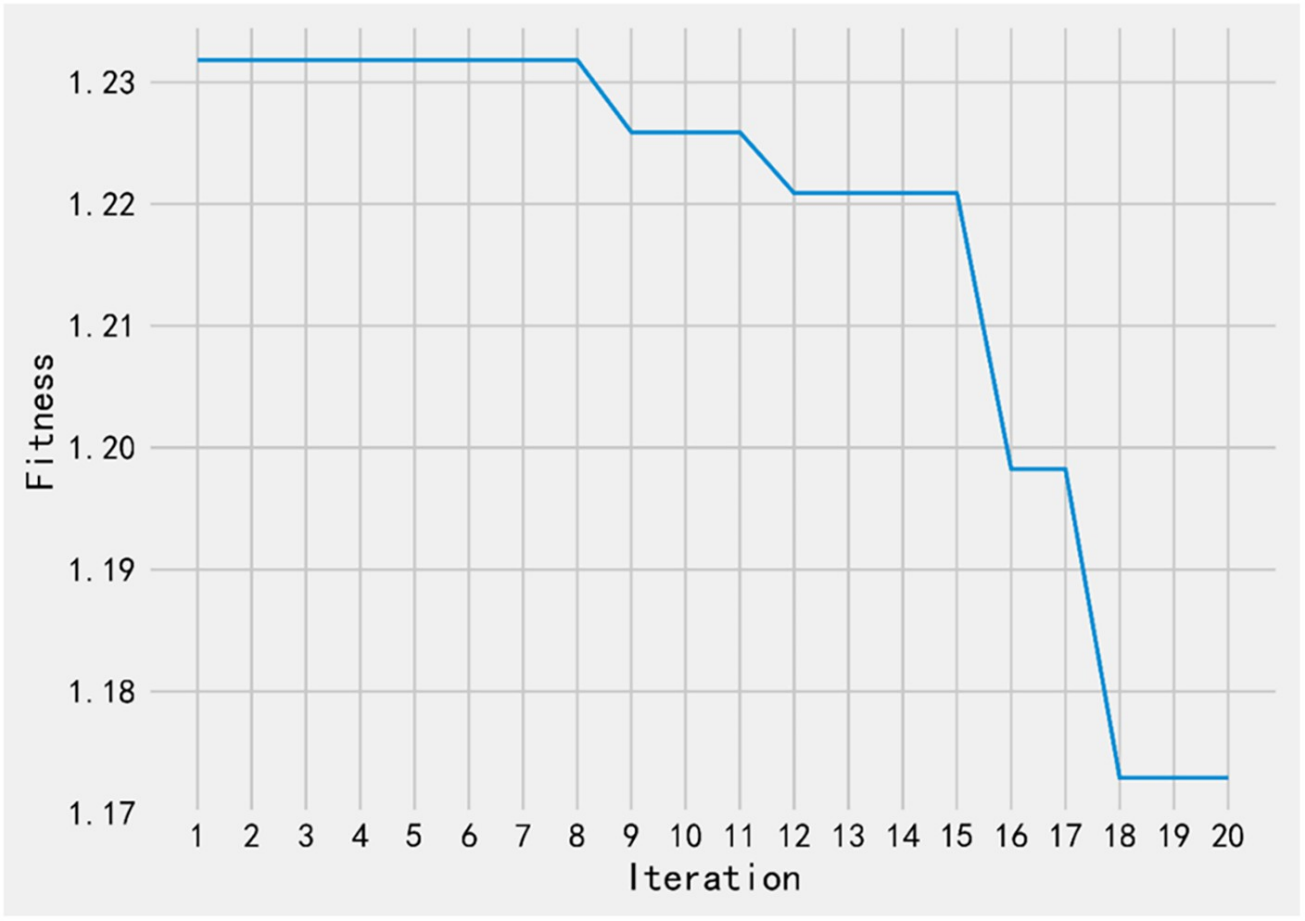
Iteration-fitness curve.

The Fig 8 shows the fitness curve plot. Fitness is a metric used to evaluate the quality of solutions in optimization algorithms. In this plot, the x-axis represents the number of iterations, and the y-axis represents the fitness values. The variable fitness in the picture is a list that contains the fitness values for each iteration. It iterates over the history and retrieves the fitness value for each iteration, storing them in the fitness list.

This fitness curve plot is used for blood glucose prediction, and the fitness value decreases as the number of iterations increases, it means that the optimization algorithm is seeking solutions with lower fitness values. In the context of blood glucose prediction, a smaller fitness value usually indicates that the predicted results are closer to or more accurate compared to the actual blood glucose values. Therefore, as the number of iterations increases, the optimization algorithm adjusts the model parameters or employs other optimization strategies to improve the prediction model for more accurate blood glucose prediction. The decreasing trend in the fitness value indicates that the algorithm is progressively approaching a better prediction model to enhance the accuracy of the predictions.

Conclusion, from the analysis of glucose data for sample 1 with the predicted glucose values at 15, 30, and 45 minutes, it can be seen from the three pieces of data predicted based on the Transformer-LSTM model are basically consistent with the original blood glucose data. The MSE values of Transformer-LSTM model in sample 1 are 1.18, 1.78, and 2.00, respectively, which are better than the transformer model. Therefore, the Transformer-LSTM model can better predict the blood glucose level.

However, there are significant disadvantages to using historical CGM data as input to the Transformer-LSTM model, such as its slowness to identify changes brought on by intrinsic and extrinsic changes, as well as potential abrupt changes in the temporal derivative of the signal. The fluctuations in blood sugar will be influenced by the patient’s basal A1C and Body Mass Index (BMI) values, and the level of activity will be influenced by the pace at which insulin is metabolised. Include quantitative variables in the model, such as insulin injections, food intake, the total number of steps taken while walking, and heart rate to further increase prediction accuracy.

In order to improve prediction accuracy and more accurately forecast the prevalence of hyperglycemia and hypoglycemia, we are now investigating various approaches to monitor patient behaviour. To enhance performance, be ready to include more variables in the model. There are still a few issues with the present body of research. There will be a delay of 5 to 15 minutes between the monitoring of the blood glucose level of the CGM itself, which is based on interstitial fluid, and the blood glucose level of capillary blood. In other words, the interstitial fluid blood sugar level can only change after the capillary blood sugar level changes, making capillary blood the most accurate measure of blood sugar. Furthermore, just the blood sugar value and CGM calibration value were examined in this study, despite the fact that these two variables are highly susceptible to change. Examples include how much insulin is administered, how many calories are ingested, how much time is spent exercising, and the impact of exercise intensity.

## Conclusions

From the simulation and prediction outcomes, we can conclude that the Transformer-LSTM model is a reliable way to predict blood glucose levels from CGM systems. Real-time prediction is another benefit of this method, which is crucial for understanding patients’ nutrition, physical activity, insulin injection, and other behaviours. The algorithm may be executed on a standard PC. Over blood sugar participants whose training set size is only 576 data for two days, the training algorithm takes 0.2 to 0.5 hours (depending on the configuration of the learning algorithm parameters). The capacity to predict blood sugar levels in real time is considerably improved by the algorithm’s quick operation. While demonstrating enhanced predictive accuracy for blood glucose levels, presents several limitations. It is heavily reliant on the quality and scope of the training data, which, being derived from only eight patients, may not represent the broader diabetic population, potentially limiting its generalizability. The model’s complexity leads to higher computational demands, which may hinder its deployment in resource-limited settings. Additionally, its performance is sensitive to hyperparameter settings, posing challenges in model tuning and increasing the risk of overfitting. There is also a concern about the model’s adaptability to new data without extensive retraining and its lack of transparency, which can be problematic in clinical environments where interpretability is crucial. These limitations underscore the need for further research to refine the model and enhance its practical utility in diverse clinical scenarios.

The future work will aim to capitalize on the foundation laid by the hybrid Transformer-LSTM model. While the current research has made significant strides in glucose level prediction, it also opens several new questions and possibilities for further exploration. Subsequent studies are encouraged to refine the algorithm, addressing the limitations encountered due to data variability and real-world application challenges. Moreover, the adaptability of the model to integrate additional physiological parameters presents a promising area for extended research. The anticipated advancements in wearable health technology and data analytics will provide fertile ground for applying and testing the model in broader contexts. Ultimately, the progression of this work is expected to not only refine diabetes management strategies but also contribute valuable insights to the broader field of predictive health analytics.
